# The Oscillatory Basis of Working Memory Function and Dysfunction in Epilepsy

**DOI:** 10.3389/fnhum.2020.612024

**Published:** 2021-01-12

**Authors:** Olivia N. Arski, Julia M. Young, Mary-Lou Smith, George M. Ibrahim

**Affiliations:** ^1^Institute of Medical Science, University of Toronto, Toronto, ON, Canada; ^2^Program in Neuroscience and Mental Health, Hospital for Sick Children Research Institute, Toronto, ON, Canada; ^3^Department of Psychology, Hospital for Sick Children, Toronto, ON, Canada; ^4^Department of Psychology, University of Toronto Mississauga, Mississauga, ON, Canada; ^5^Institute of Biomaterials and Biomedical Engineering, University of Toronto, Toronto, ON, Canada; ^6^Division of Neurosurgery, Department of Surgery, Hospital for Sick Children, University of Toronto, Toronto, ON, Canada

**Keywords:** working memory, epilepsy, neural networks, high frequency oscillations, hippocampus

## Abstract

Working memory (WM) deficits are pervasive co-morbidities of epilepsy. Although the pathophysiological mechanisms underpinning these impairments remain elusive, it is thought that WM depends on oscillatory interactions within and between nodes of large-scale functional networks. These include the hippocampus and default mode network as well as the prefrontal cortex and frontoparietal central executive network. Here, we review the functional roles of neural oscillations in subserving WM and the putative mechanisms by which epilepsy disrupts normative activity, leading to aberrant oscillatory signatures. We highlight the particular role of interictal epileptic activity, including interictal epileptiform discharges and high frequency oscillations (HFOs) in WM deficits. We also discuss the translational opportunities presented by greater understanding of the oscillatory basis of WM function and dysfunction in epilepsy, including potential targets for neuromodulation.

## Introduction

Epilepsy is a serious neurological condition that affects millions worldwide (Guerrini, 2006). While epilepsy is characterized by seizures, deficits in working memory (WM) are also pervasive (Motamedi and Meador, 2003; Holmes, 2013; Nickels et al., 2016) and associated with significant morbidity and diminished quality of life (Danguecan and Smith, 2017). The burden of WM impairment in epilepsy is underscored by the ubiquitous need for WM in adaptive functioning and cognition. In particular, WM encompasses the capacity to transiently retain information to guide goal-directed behavior (Baddeley, 1992). As such, WM is implicated in a host of higher cognitive processes and skills. Indeed, WM impairment has been associated with difficulties in academic outcomes, attention deficits, and memory impairment in children and adults with epilepsy (Fastenau et al., 2004; van Rijckevorsel, 2006; Fuentes and Kerr, 2016).

Notably, epilepsy surgery can render an individual seizure-free, but may not improve WM (Helmstaedter and Kurthen, 2001). Therefore, there is an unmet need to better understand these impairments and to develop treatments targeting WM function in individuals with epilepsy. Translational opportunities are afforded greater understanding of first, the neural substrates underlying WM function, and second, the pathophysiological mechanisms by which epileptic activity disrupts these dynamics.

Converging evidence from multiple modalities including resting-state and functional magnetic resonance imaging (rs-MRI and fMRI) and intracranial electroencephalography (EEG) demonstrates that WM relies on oscillatory interactions within and between nodes of canonical, large-scale functional networks, including the frontoparietal central executive network (FP-CEN), salience network (SN), and default mode network (DMN) (Liang et al., 2016). These oscillatory interactions occur in various frequencies, including the theta, alpha, and gamma bands. Importantly, the activity of each functional network and oscillatory frequency is specialized to subserve different subprocesses of WM (Von Stein and Sarnthein, 2000). In particular, theta oscillations in the hippocampus and prefrontal cortex (PFC) are thought to be critical to WM function, mediating the encoding, maintenance, and retrieval of stimuli as well as their governing central executive processes (Kahana et al., 2001; Sauseng et al., 2010).

The causes of WM impairment in epilepsy remain elusive and likely multifactorial. There may be primary dysfunction of underlying brain circuitry comorbid with epilepsy. Indeed, neurocognitive deficits often predate the onset of seizures and the diagnosis of epilepsy (Austin et al., 2001). Conversely, recurrent seizures, epileptiform discharges, and transient epilepsy-related events, such as high frequency oscillations (HFOs) may affect coordinated functional interactions between and within cortical regions subserving WM (Holmes and Lenck-Santini, 2006; Ewell et al., 2019). In addition, anti-epileptic drugs (AEDs), and particularly topiramate (TPM), have also been implicated in WM impairment (Kockelmann et al., 2003; Lee et al., 2003; Jansen et al., 2006; Ciantis et al., 2008; Szaflarski and Allendorfer, 2012; Yasuda et al., 2013; Tang et al., 2016; Wandschneider et al., 2017; Hu et al., 2019; Callisto et al., 2020).

The current review maps the literature pertaining to the oscillatory and large-scale network basis of WM and its impairment in epilepsy. We describe the current literature linking regional and spectral specificity to WM function. The mechanisms by which epilepsy may interfere with normative network function are summarized and explored. The current work provides a framework for WM function and dysfunction in epilepsy with a view toward expanding understanding of this fundamental process and informing future research into better treatments for affected individuals.

## Working Memory

**FIGURE 1:**
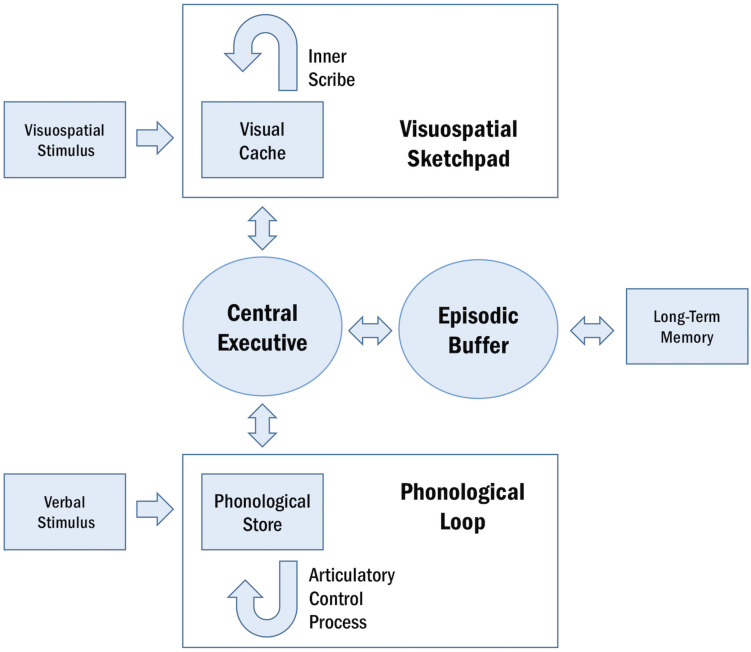
Schematic representation of the multi-component model of WM. The central executive supervises the two domain-specific subsystems, the phonological loop and the visuospatial sketchpad. Within these sub-systems, the phonological store and visual cache serve as limited-capacity stores and the articulatory control process and inner scribe rehearse information. The episodic buffer integrates information across domains and enables interactions between WM and long-term memory.

Working memory is a cognitive system that subsumes the ability to encode, maintain, manipulate, and retrieve information in a transient manner (Roux and Uhlhaas, 2014). This system is limited in capacity and operates across a range of cognitive tasks to facilitate goal-oriented behavior (Baddeley, 1992). The conceptual underpinnings of WM have been described in several models (Table 1; Baddeley and Hitch, 1974; Cowan, 1988; Ericsson and Delaney, 1999; Shah and Miyake, 1999; Repovš and Baddeley, 2006; Lovett et al., 2012). A particularly influential framework of WM is described in the multi-component model, proposed by Baddeley and Hitch (1974) and later revised by Repovš and Baddeley (2006). The multi-component model of WM assumes four functional components: the central executive, the phonological loop, the visuospatial sketchpad, and the episodic buffer (Baddeley and Hitch, 1974; Repovš and Baddeley, 2006; Figure 1).

**TABLE 1:** Summary of prominent WM models.

**Models of WM**	**Authors**	**Components**	**Access to WM information**	**Description**
Multi-component	Baddeley and Hitch, 1974; Repovš and Baddeley, 2006	Central executive Phonological loop Visuospatial sketchpad Episodic buffer	Modality-specific buffers Long-term memory activation	Central executive supervises stored information in modality-specific buffers (e.g., verbal in phonological loop and visuospatial in visuospatial sketchpad) Episodic buffer integrates information across modalities and activates long-term memory information
Embedded-processes	Cowan, 1988; Shah and Miyake, 1999	Central executive Active memory Focus of attention	Long-term memory activation	Central executive activates long-term memory information (e.g., active memory) Subset of active memory becomes focus of attention
Adaptive Control of Thought – Rational (ACT-R)	Lovett et al., 2012	Central executive Task goal	Long-term memory activation	Central executive activates long-term memory information relevant to task goals
Long-term Working Memory (LT-WM)	Ericsson and Delaney, 1999	Experience-related retrieval cues	Long-term memory activation	Experience-related retrieval cues in short term memory activate long-term memory information
				

The central executive serves as the attentional component of WM, supervising and coordinating the two subsidiary storage systems: the phonological loop and the visuospatial sketchpad. These systems are domain-specific, enabling the temporary storage and rehearsal of verbal and visuospatial information, respectively. The phonological loop and the visuospatial sketchpad are both comprised of a passive limited-capacity store (e.g., phonological store, visual cache), which holds information for a few seconds before the memory trace fades, and an active rehearsal process (e.g., articulatory control process, inner scribe), which rehearses and manipulates information (Baddeley, 1992; Logie, 2011). The episodic buffer is responsible for integrating information across domains and serves as the intermediary system between WM and long-term memory (LTM) (Baddeley and Hitch, 1974; Repovš and Baddeley, 2006).

The importance of WM is indexed by its role in supporting higher cognitive processes, including learning, memory, planning, reasoning, language comprehension, mathematical abilities, and spatial processing (Baddeley, 2003; Raghubar et al., 2010; Logie, 2011). Given the ubiquity of WM in cognitive processes, impairment in WM is debilitating and underlies a host of learning and developmental difficulties in children and can lead to functional challenges in adults (Jeffries and Everatt, 2004).

## Large-Scale Networks Subserving WM

Working memory is mediated by a distributed network of cortical and subcortical regions (Wager and Smith, 2003; Owen et al., 2005; Rottschy et al., 2012). A core WM network, comprised of frontal and parietal cortices, has been identified by several meta-analyses of neuroimaging studies (Wager and Smith, 2003; Owen et al., 2005; Rottschy et al., 2012). This frontoparietal network is associated with the central executive of WM and is known as the FP-CEN (Collette et al., 1999; Kondo et al., 2004; Li et al., 2004; Osaka et al., 2004; Sauseng et al., 2005; Palva et al., 2010). The function of the FP-CEN includes resource allocation during the simultaneous execution of two tasks (e.g., dual task coordination), modification of WM contents according to internal or external inputs (e.g., updating processes), and decision-making in the context of goal-directed behavior (Miller and Cohen, 2001; Collette and Van Der Linden, 2002). Cortical regions that are consistently implicated in the FP-CEN include the dorsolateral prefrontal cortex (dlPFC) and posterior parietal cortex (PPC)/intraparietal sulcus (IPS) (Baddeley, 2003; Seeley et al., 2007; Braunlich et al., 2015).

The FP-CEN interacts with other functional networks during WM tasks, including the SN, the dorsal attention network (DAN), and the DMN. WM demands modulate these interactions, mediating between the internally oriented activity of the DMN and the externally oriented activities of the FP-CEN, the SN, and the DAN (Liang et al., 2016).

The SN comprises the anterior insula (AI)/frontoinsular cortex and dorsal anterior cingulate cortex (dACC)/middle frontal gyrus (Braunlich et al., 2015). The SN is responsible for the detection of salient stimuli (Seeley et al., 2007). Notably, salience detection by the SN is not engendered in a task-specific manner and can encompass cognitive, homeostatic, or emotional salience (White et al., 2010). It is postulated that the FP-CEN selectively operates on salient stimuli detected by the SN (Seeley et al., 2007). These FP-CEN-mediated operations are task-specific and include maintaining and manipulating relevant stimulus representations in WM (Braunlich et al., 2015). Braunlich et al. (2015) demonstrated these dissociable WM functions of the SN and the FP-CEN using principal components analysis and fMRI during delayed-match-to-category and delayed-match-to-sample tasks. The authors identified a network comprising regions of the SN, which demonstrated a pattern of activity consistent with orienting to and processing of complex information. These regions of the SN exhibited rapid hemodynamic response peaks following stimulus onset and increased activity during conditions requiring item processing. The authors also identified a network comprising regions of the FP-CEN, which demonstrated a pattern of activity consistent with decision-making. These regions of the FP-CEN exhibited slower responses following stimulus onset and increased activity during categorization, which relies on stimulus maintenance and manipulation (Braunlich et al., 2015). Conceivably, integration of the FP-CEN and the SN is necessary for these WM-related processes, which encompass both stimulus detection and selective maintenance and manipulation of relevant stimuli (Gong et al., 2016). Indeed, resting-state coupling between core regions within the FP-CEN and the SN contributes to WM performance (Fang et al., 2016).

The DAN is comprised of important nodes in the frontal eye fields, premotor cortex, and superior parietal lobe (Braunlich et al., 2015). The DAN is closely associated with sensorimotor regions and is characterized by externally oriented activity, playing a key role in visuospatial perceptual attention (Dixon et al., 2018). The FP-CEN co-activates with the DAN during externally oriented WM tasks. Here, both networks attend to relevant stimuli in the environment (Elton and Gao, 2014).

The DMN is primarily comprised of the medial prefrontal cortex (mPFC), posterior cingulate cortex (PCC), and inferior parietal lobe (IPL) (Liang et al., 2016). The DMN is characterized by internally oriented activity and is involved in mentalizing, spontaneous cognition, and self-referential processing (Dixon et al., 2018). The DMN is negatively correlated with FP-CEN activity during WM (Clare Kelly et al., 2008) and opposing patterns of connectivity can be observed within these two networks during WM processing (Liang et al., 2016). For instance, connectivity within the FP-CEN increases with WM load, whereas connectivity within the DMN decreases with WM load (Liang et al., 2016). Interestingly, the SN facilitates switching between the FP-CEN and the DMN during WM. This switching enables the SN to allocate attentional and WM-related resources to the most salient stimuli among internal (i.e., DMN-related) and external (i.e., FP-CEN-related) events (Sridharan et al., 2008; Menon and Uddin, 2010). Notably, the SN becomes more integrated with both the FP-CEN and the DMN as WM load increases (Liang et al., 2016).

The DMN and the FP-CEN are further divided into sub-systems that are relevant to WM. The DMN is comprised of two sub-systems, the dorsal medial sub-system and the medial temporal sub-system (Andrews-Hanna et al., 2014). The dorsal medial sub-system is involved in mentalizing and social cognition and comprises the dorsal medial PFC, the temporoparietal junction, the lateral temporal cortex, and the temporal pole (Andrews-Hanna et al., 2014). The medial temporal sub-system is involved in past and future autobiographical thought, episodic memory, and contextual retrieval, and comprises the hippocampus, the parahippocampal cortex, the retrosplenial cortex, the posterior IPL and the ventromedial PFC (Andrews-Hanna et al., 2014). The medial temporal sub-system, and particularly the hippocampus, is implicated in WM.

The hippocampus plays an important role in novelty detection (Knight, 1996) and associative binding (Wallenstein et al., 1998; Yonelinas, 2013) and is consistently recruited during the encoding, maintenance, and retrieval of novel or complex information in WM (Karlsgodt et al., 2005; Cashdollar et al., 2009; Leszczynski, 2011). The activity of hippocampal neurons is thought to represent a conjunction of task-relevant features in WM, including those of a non-spatial origin (Deadwyler et al., 1996). Notably, recent findings demonstrate that hippocampal firing during WM could differentiate between success and error trials during stimulus encoding, predict workload during WM maintenance, and predict behavioral response during retrieval (Boran et al., 2019). Further evidence for the role of the hippocampus in WM derives from anatomical and behavioral dissociations, which demonstrate that lesions of the hippocampus or its extrinsic connections adversely affect WM performance (Olton and Feustle, 1981; Deadwyler et al., 1996). Additionally, the hippocampus serves as a locus of interaction between WM and LTM, supporting the encoding of information from WM into LTM and the retrieval of information from LTM into WM. Indeed, activation of the hippocampus during the maintenance of information in WM is predictive of subsequent LTM performance (Ranganath et al., 2005). Given the role of the hippocampus in associative binding and WM-LTM interactions, it is thought that the hippocampus contributes to the underlying substrate of the episodic buffer in WM.

The FP-CEN also comprises two subnetworks, FP-CEN subnetwork A and FP-CEN subnetwork B. Each subnetwork is associated with either the DAN or the DMN (Elton and Gao, 2014; Dixon et al., 2018; Murphy et al., 2020). FP-CEN subnetwork A is preferentially associated with the DMN and mainly consists of the rostrolateral PFC, middle frontal gyrus (MFG), and superior frontal gyrus (SFG) (Kam et al., 2019). During internally oriented WM tasks, the FP-CEN subnetwork A disengages with the DAN and engages with the DMN. Conversely, the FP-CEN subnetwork B is preferentially associated with the DAN and mainly encompasses the inferior frontal sulcus and the posterior aspect of the superior frontal sulcus (Kam et al., 2019). During externally oriented WM tasks, the FP-CEN subnetwork B disengages with the DMN and engages with the DAN. Together, the complementary processes of the FP-CEN subnetworks are thought to segregate external stimuli from internal trains of thought during WM (Elton and Gao, 2014; Dixon et al., 2018; Murphy et al., 2020).

**FIGURE 2:**
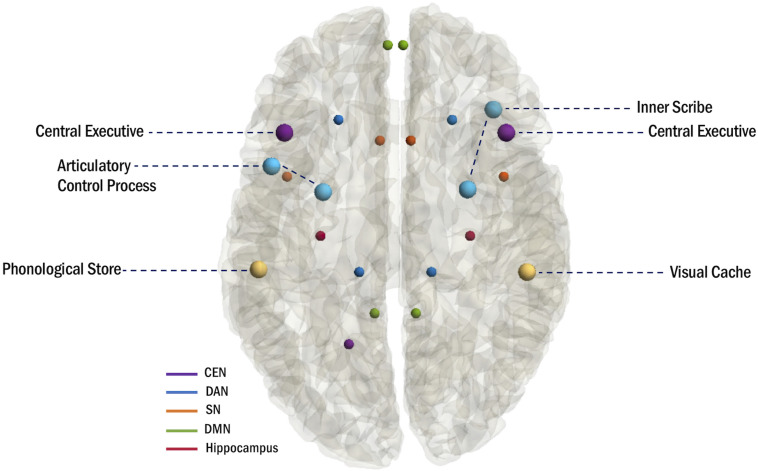
Anatomical mapping of the multi-component model of WM and hubs of WM-related networks.

In addition to the WM trends that emerge with specialization of the functional networks, material-specific lateralization has previously been demonstrated in the WM network as a collective (Sauseng et al., 2005), although these effects are less robust in children. The phonological loop is associated with left hemispheric activation (Smith et al., 1996; Sarnthein et al., 1998; Clark et al., 2001), and neuroimaging studies have identified the supramarginal gyrus (BA 40) as the phonological store and Broca’s area in the left IFG (BA 6/44) as the articulatory control process (Paulesu et al., 1993; Baddeley, 2003; Papagno et al., 2017). Conversely, the visuospatial sketchpad is associated with right hemispheric activation (Smith et al., 1996), and neuroimaging studies have identified the right inferior parietal cortex (BA 40) as the visual cache and the right premotor cortex (BA 6) and right inferior frontal cortex (BA 47) (Baddeley, 2003) as regions of the inner scribe (Baddeley, 2003; Figure 2).

## Neural Oscillations

Working memory processing depends on interactions between neuronal ensembles within WM networks (Klimesch et al., 2010). These interactions are subserved by the intrinsic oscillatory character of neuronal ensembles (Fries, 2005). As neuronal ensembles oscillate, they undergo rhythmic changes in neuronal excitability, which enable and suppress their ability to send and receive information (Buzsáki and Draguhn, 2004). For information to be propagated from one neuronal ensemble to another, the two ensembles must be excitable in the same temporal window (Fries, 2005). Neuronal coherence, the synchronization of the oscillating ensembles, facilitates information propagation by establishing a transient network with shared temporal windows for communication (Fries, 2005).

**FIGURE 3:**
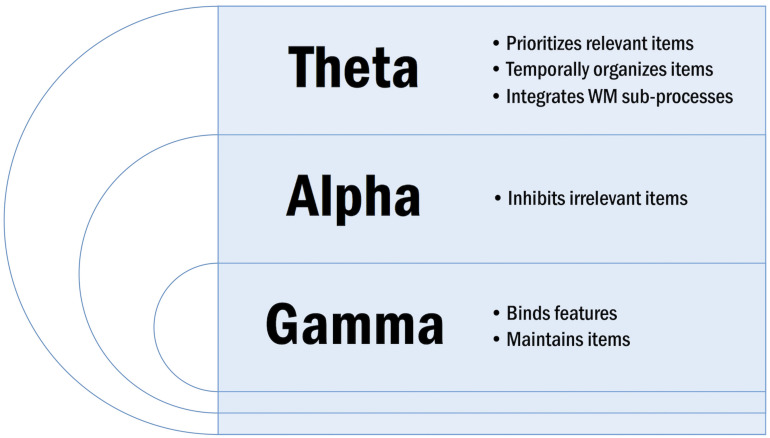
Overview of specialized oscillatory functions in WM.

Neural oscillations are subdivided into canonical bands based on frequency. These frequency bands include delta (1–4 Hz), theta (4–7 Hz), alpha (8–12 Hz), beta (15–30 Hz), and gamma (>30 Hz). Neural oscillations serve specialized functions in WM according to their frequency (Figure 3). Low frequency synchronization is observed between distant brain regions and is thought to underlie context-driven, top-down WM processes including executive control (Von Stein and Sarnthein, 2000). Conversely, high frequency synchronization is observed between local brain regions and is thought to underlie stimulus-driven, bottom-up WM processes including perception (Von Stein and Sarnthein, 2000).

Certain properties of neural oscillations can be modulated by WM, including the oscillatory amplitude and phase. The oscillatory amplitude is related to the power, which is the squared amplitude of the oscillation. Power reflects the number of neuronal units that are synchronously active and indicates the extent of task involvement: task-relevant oscillations exhibit increased amplitudes, whereas task-irrelevant oscillations exhibit decreased amplitudes (Klimesch et al., 2008). The oscillatory phase refers to the timing of neuronal excitability and is an important mechanism determining whether information is propagated within the task-relevant network. A neuron is unlikely to generate action potentials during the phase of low excitability, whereas a neuron is very likely to generate action potentials during the phase of high excitability (Fries, 2005). By extension, oscillating neuronal ensembles fire synchronously during the excitatory phase. Consequently, targeted neuronal ensembles receive the information synchronously, and information is propagated throughout the network (Klimesch et al., 2010).

In a similar vein, oscillatory phase can synchronize over large distance or modulate local oscillatory amplitude to facilitate the integration of information into WM. It is posited that phase synchronization among task-relevant brain regions can serve to integrate information across multiple spatial scales (Fries, 2005). Phase-amplitude cross-frequency coupling, wherein the amplitude of a fast oscillation is modulated by the phase of a low-frequency oscillation, is thought to integrate information across multiple temporal scales within local cortical networks (Fell and Axmacher, 2011).

## Oscillatory Basis of WM

### Theta

Theta oscillations are well-studied in the rodent brain, where they are particularly prominent in the hippocampus (Kahana et al., 2001). Hippocampal theta emerges when the rodent engages in exploratory behavior (Vanderwolf, 1969). Studies probing the medial septum-diagonal band of Broca (MS-DBB), a generator of the hippocampal theta rhythm, additionally suggest that theta oscillations in the rodent hippocampus are associated with WM function (Kahana et al., 2001). Their findings demonstrate that lesions of the MS-DBB eliminate the hippocampal theta rhythm and induce WM impairment (Olton et al., 1979; Mizumori et al., 1990), whereas addition of cholinergic agonists to the MS-DBB increases the hippocampal theta rhythm (Lawson and Bland, 1993) and enhances WM performance (Markowska et al., 1995).

The discovery of hippocampal place cells, which fire when a specific location of the environment is traversed, further facilitated investigations into the role of hippocampal theta in WM (O’Keefe and Recce, 1993). Several experiments demonstrated that theta sequences, which compress the behavioral order of place cells within a theta cycle, represent trajectories or spatial paths in the environment (Gupta et al., 2012; Wikenheiser and Redish, 2015; Kay et al., 2020). These theta sequences can vary considerably in their individual trajectory representations, wherein some sequences are confined to a narrow range around the rat’s current position while others project further beyond. It is postulated that these modulations occur according to the behavioral demands of WM. Indeed, in rats performing a value-guided decision-making task, the extent to which theta sequences projected ahead of the rat’s current position varied on a moment-by-moment basis depending on the rat’s goals (Wikenheiser and Redish, 2015). These results challenge the notion that place cells represent simple aspects of spatial and episodic memories. Conversely, it could be suggested that place cells comprise a complex system that is involved in behaviorally relevant transitions between WM and LTM.

Evidence for the functional relevance of theta oscillations in WM has since been extended to the human brain, where theta is thought to underlie WM processing in both local circuits and distributed neuronal ensembles. Previous findings demonstrate that local modulations in theta power and phase contribute to the processing and organization of WM contents, whereas long-range theta coherence integrates WM sub-processes (Sauseng et al., 2010).

#### Local Theta Activity

In local circuits, theta oscillations provide optimal neuronal ambiance for the processing of WM-related information (Sauseng et al., 2010). Notably, cortical theta power increases during WM encoding and is sustained during the retention period until retrieval (Raghavachari et al., 2001; Raghavachari et al., 2006). Theta activity additionally increases parametrically with WM load in prominent nodes of the WM network, including frontal regions of the FP-CEN and the hippocampus (Jensen and Tesche, 2002). Collectively, these synchronous theta signatures have been interpreted as a gating mechanism, enhancing attention and prioritizing relevant information during WM processing (Gevins et al., 1997; Raghavachari et al., 2001; Riddle et al., 2020).

Converging evidence suggests that local instantaneous theta phase in the hippocampus organizes WM contents. First, hippocampal theta plays a role in phase-dependent plasticity, essentially determining the likelihood of a stimulus to undergo long-term potentiation (LTP). Importantly, LTP is theorized to strengthen the connectivity between neurons and is considered a synaptic mechanism for the encoding of a stimulus into WM (Klimesch and Doppelmayr, 1996). Previously, it has been demonstrated that LTP is preferentially induced at theta rhythm periodicity (Greenstein et al., 1988) and particularly at the positive phase of the theta rhythm (Pavlides et al., 1988). Indeed, in region CA1 of the hippocampus, LTP can be induced by stimulation on the peak, but not the trough, of the theta rhythm recorded in stratum radiatum in slice preparations, urethane-anesthetized rats, and awake rats (Hölscher et al., 1997; Hyman et al., 2003).

Second, hippocampal theta plays a role in phase-dependent coding of information. In rodents, spatial WM information is represented by the alignment of hippocampal place cell firing to specific phases of theta band activity (O’Keefe and Recce, 1993). In humans, Hasselmo et al. (2002) have proposed a model wherein hippocampal theta phase segregates encoding and retrieval phases in WM. In this model, WM encoding is associated with the trough of theta recorded at the hippocampal fissure – equivalent to the peak of theta recorded in stratum radiatum – when there is strong synaptic input from the entorhinal cortex into the stratum lacunosum-moleculare. Here, there is weak synaptic input from region CA3 of the hippocampus, however these same synapses show a strong capacity for LTP. Collectively, these phenomena enable the encoding of afferent information from the entorhinal cortex, while preventing interference from previously encoded information arising from region CA3 of the hippocampus. Conversely, retrieval is associated with the peak of theta recorded at the hippocampal fissure – equivalent to the trough of theta recorded in stratum radiatum – when there is relatively weak synaptic input from the entorhinal cortex into the stratum lacunosum-moleculare (Hasselmo et al., 2002). Here, there is strong synaptic input from region CA3 of the hippocampus, however these same synapses show a weaker capacity for LTP and tend to undergo long-term depression (LTD). Collectively, these phenomena enable retrieval of previously encoded information, while preventing further encoding of retrieval activity. The model proposed by Hasselmo et al. (2002) has been corroborated by evidence which demonstrates that theta oscillations exhibit a phase difference of 180° between WM encoding and retrieval (Rizzuto et al., 2006).

Third, hippocampal theta is phase-locked to WM-related stimuli (Givens, 1997; Tesche and Karhu, 2000; Rizzuto et al., 2003). Phase-locking occurs when the presentation of a WM-related stimulus causes the phases of an ongoing hippocampal theta oscillation to re-align or reset. In a seminal study, Givens (1997) demonstrated that phase resetting of the hippocampal theta rhythm in rodents occurs exclusively in response to WM-related stimuli, which are actively processed in the hippocampus, and not in response to reference memory-related stimuli. Givens (1997) hypothesized that this resetting phenomenon allows the hippocampus to experience a wave of depolarization at precisely the time that relevant sensory stimuli arrive in the hippocampus from the entorhinal cortex. Specifically, the phase-locking of theta oscillations would allow for later arriving and more highly processed sensory information to be potentiated or reverberated through several autoassociative theta cycles, which would ultimately facilitate the encoding of sensory information into WM. McCartney et al. (2004) corroborated this hypothesis, demonstrating that phase resetting of the hippocampal theta rhythm promotes optimal conditions for WM-related stimuli to be encoded and potentiated into memory.

Phase resetting has since been demonstrated in humans with similar manifestations, wherein the presentation of a behaviorally relevant stimulus in WM, such as a list item or probe, is followed by phase-locking in neocortical (Rizzuto et al., 2003) and hippocampal (Tesche and Karhu, 2000; Kleen et al., 2016) oscillations. This phase-locking has been reported in various frequencies, including delta, theta, and alpha bands (Rizzuto et al., 2003; Kleen et al., 2016). Notably, Kleen et al. (2016) observed that the degree of phase resetting in delta, theta, and alpha bands correlated with WM performance.

Interestingly, emerging evidence suggests that the properties of phase-locking in the theta band during WM are dependent on item content and load (Kamiński et al., 2020). In low loads, neurons phase-lock to the theta rhythm only when their preferred item is in WM, whereas in higher loads, the phase of the theta rhythm that neurons phase-lock to depends on whether the preferred item is in WM (Kamiński et al., 2020). These findings describe a putative mechanism by which theta phase could orchestrate hippocampal neural activity to successfully maintain multiple items in WM (Kamiński et al., 2020).

#### Long-Range Theta Coherence

Long-range theta coherence is thought to integrate WM sub-processes (Sauseng et al., 2010). Synchronous theta activity is consistently reported between frontal and temporo-parietal regions during the encoding, maintenance, and retrieval of WM information (Sarnthein et al., 1998; Sauseng et al., 2004; Wu et al., 2007). Furthermore, this oscillatory phenomenon has material-specific manifestations. For instance, Sauseng et al. (2004) reported that the encoding of visual information is characterized by theta coupling between the dlPFC and right posterior temporal regions, whereas during retrieval of verbal and visuospatial information, theta coupling occurs between prefrontal and bilateral temporo-parietal regions. Sarnthein et al. (1998) reported similar findings during the retention of verbal and visuospatial information, wherein theta coupling was observed between the PFC and posterior association cortex. Notably, interregional theta synchronization could play a role in integrating multi-modal information. Wu et al. (2007) used EEG to investigate phase synchronization in a WM task, wherein participants retained verbal information (e.g., letters), visuospatial information (e.g., locations), or bound information from both modalities (e.g., letters and locations). The authors found that theta phase synchronization increased between bilateral frontal regions and between the left frontal and right temporal-parietal regions during the maintenance of bound verbal and visuospatial information relative to segregated information (Wu et al., 2007). In these collective findings, long-range theta coherence between frontal and temporo-parietal regions likely serves to integrate processes that underly the storage of sensory information (e.g., temporo-parietal activity) and processes that underly the maintenance and updating of current relevant information (e.g., frontal activity) (Sarnthein et al., 1998).

Experiments in rodents support the postulation that theta coherence between the PFC and the hippocampus supports WM performance (Hyman et al., 2005; Jones and Wilson, 2005a,b; Kleen et al., 2011). In particular, mPFC neurons can be entrained to the hippocampal theta rhythm, and this entrainment is implicated in learning and memory during WM processing. In fact, mPFC cells that are actively involved in behavioral tasks are predisposed to fire entrained to the hippocampal theta rhythm (Hyman et al., 2005). Indeed, it has previously been demonstrated that a subset of neurons in the mPFC that predict the turn choices of a rat during a WM task are more strongly phase-locked to hippocampal theta than non-predicting cells (Fujisawa and Buzsáki, 2011). Furthermore, it has been observed that the most robust instances of mPFC phase precession coincide with enhanced CA1-mPFC coherence and occur during behavioral epochs, which demand the transfer of information from CA1 to mPFC (Jones and Wilson, 2005a).

Moreover, long-range theta synchronization between frontal and temporo-parietal regions could reflect central executive functions mastering WM sub-components (Sauseng et al., 2010). In this framework, theta coupling would enable the frontal central executive to access posterior, modality-specific storage sub-systems during WM (Sauseng et al., 2010). In line with this postulation, Sauseng et al. (2005) reported increased theta coupling between fronto-parietal regions with increasing central executive demands. Furthermore, there is substantial evidence for long-range theta coherence during attentionally demanding, central executive-dependent tasks, including between the FP-CEN subnetwork A and the DMN during internal attention (Kam et al., 2019) and within the FP-CEN during mental arithmetic, which requires mental manipulation of information and continuous updating of the WM store (Sauseng et al., 2010). Further support for this postulation derives from recent evidence which demonstrates that communication between the medial temporal lobe (MTL) and the PFC is bi-directional (Johnson et al., 2018). This bi-directional communication is facilitates central executive functions in WM by coordinating PFC-guided parallel processing of incoming information and MTL-dependent information prioritization in space and time (Johnson et al., 2018).

### Alpha

Alpha oscillations are prominent in sensory regions and the thalamus (Roux and Uhlhaas, 2014). Alpha synchronization is consistently observed in posterior regions during the maintenance of WM (Jensen et al., 2002; Klimesch et al., 2010; Bonnefond and Jensen, 2012; Riddle et al., 2020), and this activity increases parametrically with WM load (Jensen and Tesche, 2002). Recently, these findings have been recapitulated in a larger-scale WM network, wherein load-dependent alpha-theta coupling was observed between the hippocampus and parietal scalp electrodes during WM maintenance (Boran et al., 2019). It is posited that these collective alpha signatures reflect functional inhibition of task-irrelevant brain regions (Jensen et al., 2002; Jokisch and Jensen, 2007; Klimesch et al., 2010; Bonnefond and Jensen, 2012; Roux and Uhlhaas, 2014; Riddle et al., 2020). Indeed, studies probing visuospatial attention and WM demonstrate that attention directed toward one visual hemifield is expressed as an ipsilateral increase and/or a contralateral decrease of posterior alpha power (Medendorp et al., 2007). Interestingly, recent evidence suggests that the inhibitory function of alpha applies to both exogenous and endogenous information; irrelevant exogenous information is suppressed from being encoded into WM, whereas endogenous information that is already encoded into memory is suppressed when it is no longer relevant to guide future behavior (Riddle et al., 2020).

Conversely, alpha desynchronization reflects a release from functional inhibition and is often associated with activation processes related to attention (Michels et al., 2008). For instance, stimulus monitoring during WM is characterized by alpha desynchronization in nodes of the DAN. This desynchronization facilitates external attention, allowing regions of the DAN to engage in neural processing that enables the detection of relevant stimuli in the environment (Cona et al., 2020). On a similar vein, alpha desynchronization is thought to support the attentional demands of the WM central executive (Michels et al., 2008). Indeed, short-range alpha coherence between frontal regions in the FP-CEN decreases with central executive needs, allowing these regions to fulfill increased attentional demands (Sauseng et al., 2005).

### Gamma

Gamma oscillations are detectable in cortical regions and some subcortical regions (Roux and Uhlhaas, 2014). Gamma synchronization occurs in local circuits and has previously been associated with perception and feature integration (Singer and Gray, 1995; Von Stein and Sarnthein, 2000). It is further posited that these gamma signatures could reflect the neuronal correlate of maintained WM representations (Jokisch and Jensen, 2007). In line with this postulation, sustained gamma oscillatory activity has been reported during the retention of various domains of stimuli, including visual, visuospatial, auditory, and somatosensory information (Roux and Uhlhaas, 2014). Moreover, gamma oscillations synchronize with increasing WM load, and this activity occurs in the hippocampus and key nodes of the FP-CEN that are integral to WM maintenance (Howard et al., 2003; Palva et al., 2010, 2011; Van Vugt et al., 2010; Roux et al., 2012).

Importantly, gamma oscillations can couple with theta or alpha oscillations to form a distinct oscillatory code that is specialized for a type of WM information. A theta-gamma code is thought to underlie the maintenance of sequential WM items and be related to a frontohippocampal network (Axmacher et al., 2010; Roux and Uhlhaas, 2014). In a framework proposed by Lisman and Idiart, individual WM items are represented by single gamma periods, which are nested into a single theta period. Here, the sequence of WM items is coded via the phase relationship between theta and gamma. Corroborating evidence of a theta-gamma code has been reported by Axmacher et al. (2010), who demonstrate that the maintenance of multiple items in WM is accompanied by load-dependent theta-gamma coupling in the hippocampus.

Additionally, an alpha-gamma code is thought to underlie the maintenance of sensory-spatial WM items. Roux and Uhlhaas (2014) propose that this oscillatory code is related to a thalamocortical network, comprising the PFC, parietal cortex, and thalamus. In this framework, gamma oscillations underlie the maintenance and read-out of relevant WM items, whereas alpha oscillations are involved in the inhibition of task-irrelevant WM items. In contrast to theta-gamma interactions, there is little evidence that directly portrays this alpha-gamma activity. However, Roux et al. (2012) review convincing evidence, which demonstrates that if WM contents are changed from multiple sequentially ordered items to discrete visual or spatial information, theta activity is replaced by alpha activity.

## WM in Epilepsy

Working memory impairment is well-documented in both children (Hernandez et al., 2002; Myatchin and Lagae, 2011; Sherman et al., 2012; Braakman et al., 2013; Longo et al., 2013) and adults (Hermann and Seidenberg, 1995; Black et al., 2010; Mwangala et al., 2018) with epilepsy. WM impairment is common across epilepsy types, manifesting in primary generalized epilepsies (Swartz et al., 1994), temporal lobe epilepsy (TLE) (Stretton and Thompson, 2012), and frontal lobe epilepsy (FLE) (Swartz et al., 1994). In both childhood and adulthood epilepsies, several factors are associated with greater risk of WM impairment, including younger age at seizure onset, longer duration of epilepsy, higher seizure frequency, and AED polytherapy (Meador, 2002; Black et al., 2010; MacAllister et al., 2012; Sherman et al., 2012; Fuentes and Kerr, 2016). Nonetheless, individuals with recently diagnosed epilepsies or well-controlled, benign epilepsies are also vulnerable to WM impairment (Myatchin and Lagae, 2011). In childhood epilepsies, WM impairment is a key feature distinguishing the cognitive profiles of children with epilepsy from healthy controls on formal intelligence tests (Sherman et al., 2012). Furthermore, WM impairment is implicated in all areas of academic achievement (Fastenau et al., 2004; Fuentes and Kerr, 2016). In adulthood epilepsies, the most frequently reported cognitive complaints are related to WM processing as well as mental slowness, attention deficits, and memory impairment (van Rijckevorsel, 2006). Notably, subjective cognitive impairment is associated with objective measures in WM and no other cognitive domains (Feldman et al., 2018).

## WM Networks in Epilepsy

Normative WM networks are perturbed in epilepsy. These perturbations are marked by changes in functional connectivity between regions in the WM network. It is posited that hypoconnectivity within the epileptic WM network indicates network dysfunction, whereas hyperconnectivity has previously been interpreted as an indicator of network dysfunction, network reorganization, or a compensatory mechanism (Gutierrez-Colina et al., 2020). In the literature, studies probing network changes in epilepsy report heterogenous findings.

In resting-state fMRI, hypoconnectivity has been observed between the FP-CEN and the SN, as well as within the FP-CEN, the SN, and cerebellar regions (Gutierrez-Colina et al., 2020). Conversely, hyperconnectivity has been reported within frontal regions and also between interhemispheric frontal and parietal regions in the same modality (Gutierrez-Colina et al., 2020). In task-based measures, hypoconnectivity has been observed in a specific subset of frontal lobe connections in children with FLE, including local connections (e.g., within the frontal lobe) and distant connections (e.g., between the anterior cingulate cortex of the SN and the superior parietal lobe of the DAN) (Braakman et al., 2013). Additionally, children with TLE show less activation in the FP-CEN (Oyegbile et al., 2018) and less de-activation in the DMN relative to healthy controls (Oyegbile et al., 2019). Importantly, these collective resting-state and task-based signatures have been associated with worse measures of WM, suggesting that aberrant connectivity may underpin WM deficits in epilepsy.

## Pathophysiological Mechanisms of WM Impairment in Epilepsy

A multitude of factors likely contributes to WM impairment in epilepsy, including the epileptogenic substrate, recurrent seizures, interictal epileptic activity, and AED therapy (Motamedi and Meador, 2003; Sherman et al., 2012; Ibrahim et al., 2014). Here, the putative contributions of interictal epileptic activity and AED therapy will be reviewed.

### Interictal Epileptiform Discharges

Interictal epileptiform discharges (IEDs) are spikes, sharp waves, or spike-wave complexes that occur without observed clinical seizures (Noachtar and Rémi, 2009). IEDs can induce a phenomenon known as transient cognitive impairment (TCI). In TCI, the occurrence of an IED is accompanied by a transient disturbance in neural processing and cognitive function (Aarts et al., 1984; Binnie, 1993). Previous works suggest that WM is particularly vulnerable to IED-induced TCI (Hutt and Gilbert, 1980; Aarts et al., 1984; Binnie et al., 1987). This increased vulnerability could be attributed to the characteristically high information processing demands of WM (Aldenkamp and Arends, 2004).

Transient cognitive impairment is demonstrable in 50% of patients who exhibit IEDs during a WM task (Binnie, 1993). The nature of the WM impairment is dependent upon where the IED occurs in the brain (Holmes, 2014). Material-specific deficits have previously been reported, wherein right-hemispheric IEDs are associated with errors in non-verbal WM tasks and left-hemispheric IEDs are associated with errors in verbal WM tasks (Aarts et al., 1984; Binnie et al., 1987). Interestingly, IEDs occurring in the mesial temporal lobe have been associated with a 6% decline in WM performance (Krauss et al., 1997). However, it is to be noted that even the occurrence of local IEDs could have widespread effects in the brain. For instance, IEDs could propagate from the hippocampus to the PFC and prevent synchronization between these structures during key WM steps (Corkin, 2001). In a similar vein, it has recently been demonstrated that hippocampal IEDs induce spindles in the mPFC and that both IED frequency and coupling with mPFC spindles are correlated with the degree of memory impairment (Gelinas et al., 2016).

Working memory impairment is additionally dependent upon the timing of the IED during WM. For instance, Kleen et al. (2010, 2013) observed that hippocampal IEDs were related to decrements in WM retrieval, but not encoding, in both rats and humans. Given that WM retrieval is dependent upon the functioning and integrity of intrahippocampal circuitry, this WM sub-process could be particularly sensitive to disruption following hippocampal IED. Conversely, WM encoding could be buffered by other cortical structures, such as the PFC or primary sensory areas, the latter of which could hold lingering stimulus representations (Kleen et al., 2013).

Studies leveraging intracranial EEG have helped elucidate putative mechanisms of IED-induced WM impairment. The occurrence of an IED in the hippocampus is followed by a sustained reduction of action potentials for a period of up to 2 s. Moreover, when IEDs occur in flurries, action potential firing could be reduced for a period of up to 6 s (Zhou et al., 2007). This IED-induced inhibitory wave disrupts WM-related oscillatory signatures in the hippocampus, resulting in reductions of hippocampal gamma (Urrestarazu et al., 2006) and theta power (Fu et al., 2018). By extension, IEDs could conceivably disrupt the organization and functioning of WM networks. Indeed, large-scale network changes precede (Ibrahim et al., 2014) and follow IEDs (Lengler et al., 2007; Ibrahim et al., 2014; Dahal et al., 2019). Moreover, the vulnerability of network topologies to IEDs has previously been associated with worse neurocognitive outcomes (Ibrahim et al., 2014).

### High Frequency Oscillations

Pathological high frequency oscillations (HFOs) are transient events detectable in the interictal EEG (Engel et al., 2009). These phenomena have recently emerged as biomarkers of epileptogenicity (Jacobs et al., 2012). Further evidence suggests that HFOs may perturb neural processing that is critical to WM, akin to the effects of IEDs (Ewell et al., 2019; Liu and Parvizi, 2019; Sun et al., 2020). The neurophysiological underpinnings of HFO-induced WM impairment remain elusive. However, it is conceivable that their pathophysiological mechanisms resemble those of IEDs, encompassing disruptions of oscillatory network activity (Brennan and Ahmed, 2019).

Indeed, HFOs have been shown to disrupt hippocampal network function in a rodent model of epilepsy (Ewell et al., 2019). To probe the effects of HFOs on the hippocampal network, Ewell et al. (2019) leveraged high-density single unit and local field potential recordings from the hippocampi of behaving rats with and without chronic epilepsy. The authors reported that the occurrence of HFOs in the epileptic hippocampus impaired spatial coding during foraging behavior via the induction of spurious, uninformative action potentials and the transient reduction of hippocampal theta power (Ewell et al., 2019).

Evidence of HFO-induced TCI has recently been extended to humans, where it has been demonstrated that the occurrence of HFOs in epileptic tissue results in a cognitive refractory state (Liu and Parvizi, 2019). Liu and Parvizi (2019) leveraged intracranial EEG recordings from non-lesional epileptic tissue to probe the effects of HFOs on stimulus-locked physiological activity. The authors observed normative physiological responses to relevant cognitive stimuli in epileptic tissue. However, these physiological responses were more likely to be “seized” (i.e., delayed or missed) when HFOs occurred around the onset of the relevant cognitive stimulus (850–1050 ms prior to stimulus onset, until 150–250 ms following stimulus onset). Furthermore, HFOs in the MTL affected memory performance. The authors concluded that a relevant cognitive stimulus will fail to activate epileptic tissue if it arrives within a shared temporal window as an HFO; this failure to activate the tissue is the pathophysiological mechanism underlying the impaired memory performance (Liu and Parvizi, 2019).

Notably, it has recently been demonstrated that the removal of HFO-generating tissue is associated with post-operative cognitive improvement in children with epilepsy (Sun et al., 2020). To probe whether the number of HFOs in pre- and post-resection intracranial EEG was associated with clinically relevant cognitive improvement, Sun et al. (2020) retrospectively reviewed intracranial EEG data and neuropsychological scores from children who were seizure free after epilepsy surgery. The authors found that children with clinically relevant, improved intelligence quotients (IQ) had significantly more HFOs in the resected tissue and fewer HFOs in the post-resection intracranial EEG relative to children with clinically irrelevant improvements (Sun et al., 2020).

### AEDs

Another potential cause of impaired WM in patients with epilepsy is treatment with AEDs. AED treatment in epilepsy protects against seizures by modulating neuronal excitability (Rogawski and Löscher, 2004). AEDs generally provide satisfactory control of seizures for most patients (Rogawski and Löscher, 2004), however AED tolerability within the cognitive domain is variable: some agents result in psychomotor slowing, reduced vigilance, and WM impairment (Motamedi and Meador, 2004), whereas others are associated with enhanced WM (Eddy et al., 2011). Two AEDs that have consistently been implicated in WM function are TPM and levetiracetam (LEV).

Topiramate is an AED with multiple mechanisms of action, including the potentiation of GABAergic neurotransmission, inhibition of voltage-dependent sodium and calcium currents, blockage of AMPA/KA receptors, and enhancement of potassium currents (Czapinski et al., 2005). TPM therapy has previously been associated with WM impairment (Kockelmann et al., 2003; Lee et al., 2003; Jansen et al., 2006; Ciantis et al., 2008; Szaflarski and Allendorfer, 2012; Yasuda et al., 2013; Tang et al., 2016; Wandschneider et al., 2017; Hu et al., 2019; Callisto et al., 2020). WM performance deteriorates following initiation of TPM therapy (Hyman et al., 2003), and discontinuation of TPM therapy is associated with significant improvements in WM (Kockelmann et al., 2003; Lee et al., 2003). Functional neuroimaging studies report that TPM therapy is associated with decreased activation in FP-CEN frontal regions (Jansen et al., 2006; Ciantis et al., 2008; Szaflarski and Allendorfer, 2012; Wandschneider et al., 2017) and impaired deactivation of regions in the DMN during WM (Szaflarski and Allendorfer, 2012; Yasuda et al., 2013; Tang et al., 2016; Wandschneider et al., 2017). Recent evidence suggests that the severity of TPM-related WM impairment is modulated by TPM plasma concentration and WM capacity (Callisto et al., 2020). Interestingly, WM capacity is negatively associated with the WM-load modulation of alpha power, and the administration of TPM weakens this association (Hu et al., 2019).

Levetiracetam is an AED with a unique mechanism of action, which involves binding a protein known as synaptic vesicle protein 2A (SV2A) (Lynch et al., 2004), which mediates calcium-dependent vesicular neurotransmitter release (Nowack et al., 2010). LEV is derived from piracetam, a drug that seems to improve learning, memory, and attention (Genton and Van Vleymen, 2000). Piracetam has previously been used to treat memory disturbances in age-related cognitive function or decline (Israel et al., 1994) and aphasia (Huber et al., 1997). It is posited that piracetam derivatives could influence the metabolism of cortical regions responsible for language and attention (Piazzini et al., 2006). Indeed, LEV therapy has previously been associated with improvement in verbal fluency (Piazzini et al., 2006) and WM (López-Góngora et al., 2008; Operto et al., 2019). Interestingly, LEV treatment decreases centrotemporal spike-associated activation in Rolandic epilepsy (Zhang et al., 2018), and neuroimaging findings demonstrate that LEV therapy is associated with restoration of normative activation patterns during WM (Wandschneider et al., 2014).

The mechanism by which LEV supports WM function is unclear. Notably, both LEV and piracetam belong to the pyrrolidine class of drugs, which exhibit low toxicity, protect against brain insults, and enhance the efficacy of higher integration mechanisms in the brain (Schindler, 1989). Conceivably, LEV could enhance the capacity of functionally compromised cortical regions to be reintegrated into the WM network (Piazzini et al., 2006).

## Discussion

In summary, WM is a critical component of cognition that is supported by dynamic oscillatory interactions between distributed cortical and subcortical regions. WM impairment is a pervasive co-morbidity of epilepsy that is likely influenced by pathological disturbances in WM network function. As reviewed, converging evidence suggests that there are disturbances to the FP-CEN, the SN, and the DMN (i.e., “the triple network”) in epilepsy. Notably, disturbances of the triple network have been associated with several psychiatric and learning disorders that are characterized by WM impairment, including depression, ADHD, schizophrenia, autism, and frontotemporal dementia (Gürsel et al., 2018). These findings lend credence to the notion that these disorders, and their cognitive co-morbidities, are underpinned by disturbances in widespread networks.

The current clinical benchmark of successful treatment of epilepsy is seizure-freedom. However, individuals may continue to suffer from WM impairments after being rendered seizure-free. IEDs and HFOs are putative pathophysiological mechanisms by which WM networks and their oscillatory signatures continue to be perturbed. Future work should aim to further elucidate the neurophysiological underpinnings of these disturbances, as these findings would provide insight for interventions that could target WM function in epilepsy. Neuromodulatory treatments aimed at suppressing these aberrant signatures and restoring normative network dynamics could be especially promising in this objective. Furthermore, IEDs and HFOs recorded in intracranial EEG could serve as biomarkers in the prediction and understanding of cognitive outcome after epilepsy surgery (Sun et al., 2020).

## Author Contributions

OA and GI prepared and revised the manuscript. JY and M-LS revised the manuscript. All authors contributed to the article and approved the submitted version.

## Conflict of Interest

The authors declare that the research was conducted in the absence of any commercial or financial relationships that could be construed as a potential conflict of interest.

## Abbreviations

AEDs: anti-epileptic drugs
AI: anterior insula
dACC: dorsal anterior cingulate cortex
DAN: dorsal attention network
dlPFC: dorsolateral prefrontal cortex
DMN: default mode network
EEG: electroencephalography
fMRI: functional magnetic resonance imaging
FP-CEN: frontoparietal central executive network
FLE: frontal lobe epilepsy
HFOs: high frequency oscillations
IEDs: interictal epileptiform discharges
IPL: inferior parietal lobe
IPS: intraparietal sulcus
IQ: intelligence quotient
LEV: levetiracetam
LTD: long-term depression
LTM: long-term memory
LTP: long-term potentiation
MFG: middle frontal gyrus
mPFC: medial prefrontal cortex
MS-DBB: medial septum-diagonal band of Broca
MTL: medial temporal lobe
PCC: posterior cingulate cortex
PFC: prefrontal cortex
PPC: posterior parietal cortex
rs-MRI: resting-stating magnetic resonance imaging
SFG: superior frontal gyrus
SN: salience network
SV2A: synaptic vesicle protein 2A
TCI: transient cognitive impairment
TLE: temporal lobe epilepsy
TPM: topiramate
WM: working memory.
